# Pharmacokinetic modeling optimizes inhibition of the ‘undruggable’ EWS-FLI1 transcription factor in Ewing Sarcoma

**DOI:** 10.18632/oncotarget.1495

**Published:** 2013-11-11

**Authors:** Sung-Hyeok Hong, Sarah. E. Youbi, S. Peter Hong, Bhaskar Kallakury, Phillip Monroe, Hayriye V Erkizan, Julie S. Barber-Rotenberg, Peter Houghton, Aykut Üren, Jeffrey A. Toretsky

**Affiliations:** ^1^ Department of Oncology, Lombardi Comprehensive Cancer Center, Georgetown University, Washington, DC, USA; ^2^ Battelle Memorial Institute, Health and Life Sciences, Columbus, OH, USA; ^3^ Department of Pathology, Lombardi Comprehensive Cancer Center, Georgetown University, Washington, DC, USA; ^4^ Nationwide Children's Research Institute, Center for Childhood Cancer, Columbus, USA

**Keywords:** YK-4-279, EWS-FLI1, Ewing Sarcoma, rat xenograft, pharmacokinetic modeling

## Abstract

Transcription factors have long been deemed ‘undruggable’ targets for therapeutics. Enhanced recognition of protein biochemistry as well as the need to have more targeted approaches to treat cancer has rendered transcription factors approachable for therapeutic development. Since transcription factors lack enzymatic domains, the specific targeting of these proteins has unique challenges. One challenge is the hydrophobic microenvironment that affects small molecules gaining access to block protein interactions. The most attractive transcription factors to target are those formed from tumor specific chromosomal translocations that are validated oncogenic driver proteins. EWS-FLI1 is a fusion protein that results from the pathognomonic translocation of Ewing sarcoma (ES). Our past work created the small molecule YK-4-279 that blocks EWS-FLI1 from interacting with RNA Helicase A (RHA). To fulfill long-standing promise in the field by creating a clinically useful drug, steps are required to allow for *in vivo* administration. These investigations identify the need for continuous presence of the small molecule protein-protein inhibitor for a period of days. We describe the pharmacokinetics of YK-4-279 and its individual enantiomers. *In vivo* studies confirm prior *in vitro* experiments showing (S)-YK-4-279 as the EWS-FLI1 specific enantiomer demonstrating both induction of apoptosis and reduction of EWS-FLI1 regulated caveolin-1 protein. We have created the first rat xenograft model of ES, treated with (S)-YK-4-279 dosing based upon PK modeling leading to a sustained complete response in 2 of 6 ES tumors. Combining laboratory studies, pharmacokinetic measurements, and modeling has allowed us to create a paradigm that can be optimized for *in vivo* systems using both *in vitro* data and pharmacokinetic simulations. Thus, (S)-YK-4-279 as a small molecule drug is ready for continued development towards a first-in-human, first-in-class, clinical trial.

## INTRODUCTION

Ewing Sarcoma (ES) is a rare cancer of bone or soft tissue that affects 3 people per million each year, largely adolescents between the ages of 10 and 25 [1], Those diagnosed with local disease experience a 5-year survival rate of 73%, while those with metastatic disease have a survival of 20–30% after 2–3 years [2–4]. *EWSR1-FLI1* is the product of a t(11;22)(q24;q12) chromosomal translocation leading to a fusion protein, EWS-FLI1, containing the amino half of the EWS protein and the carboxy-half of *ets* family transcription factor FLI1, including its DNA binding domain [5]. ES oncogenesis is driven by the chimeric transcription factor, EWS-FLI1, which is only present in the tumor cells of patients. The interaction between EWS-FLI1 and RNA Helicase A (RHA) is critical for driving ES transformation [6], and the small molecule YK-4-279 disrupts the interaction between EWS-FLI1 and RHA leading to apoptotic cell death [7].

Transcription factors have largely been considered ‘undruggable’ in general, given both their lack of enzymatic activity and intrinsically disordered domains that challenge crystallization [8]. EWS-FLI1 is a particularly diffcult oncogene to target since it is predicted to be an intrinsically disordered protein (IDP) [9–11]. EWS-FLI1 has a highly conserved *ets* DNA-binding domain in the FLI1 portion of the protein, which constitutes the only structured region [9, 12]. IDP-drug interactions are generally highly specific, however, with low affinity lending to reversible binding between the protein and drug [13–15]. Pharmaceutical development paradigms have proclaimed the need for nanomolar binding drug to achieve clinical utility, however, many current drugs show micromolar affinity for their known targets [16]. Achieving nanomolar binding of a small molecule to a protein may not always be possible while maintaining drug-like molecular weight and solubility [17, 18]. In particular, small molecule inhibitors of protein-protein interactions may only achieve effective concentrations in the micromolar range [19]. EWS-FLI1 is recognized as an ideal anticancer target, yet in part due to these perceived biochemical challenges, no small molecule has entered the clinic that directly targets this key oncoprotein.

Our previous work demonstrated (S)-YK-4-279 as the active enantiomer of YK-4-279 in multiple molecular and cell biology assays [20]. This current research resolves some of the challenges in the preclinical development of the EWS-FLI1 inhibitor (S)-YK-4-279. Using an iterative approach between *in vitro* cell line studies and pharmacokinetics in two species, we explored various dosing regimens and chose a continuous infusion model for in vivo efficacy testing. (S)-YK-4-279 reduced caveolin-1, an EWS-FLI1 regulated target gene, *in vivo*. Novel rat xenograft of ES treated with continuous infusion therapy demonstrated both achievable delivery and promising clinical efficacy.

## RESULTS

### YK-4-279 induced cell death requires constant exposure over time

**Figure 1:**
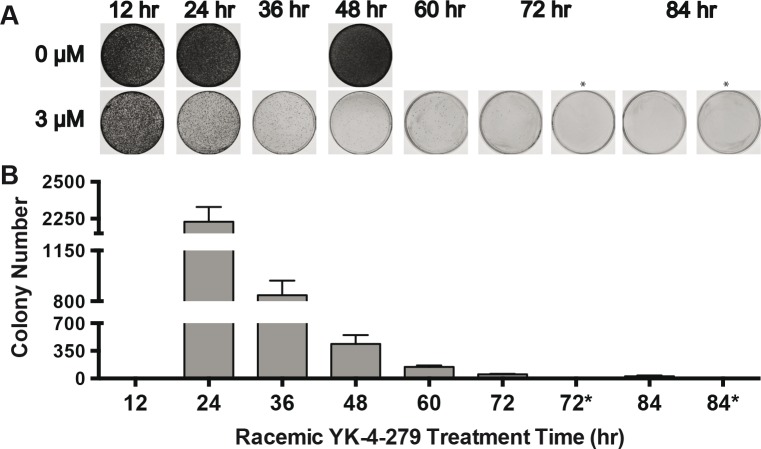
Exposure time to YK-4-279 is a critical variable to eliminate ES clonogenicity (A) A4573 cells were replated following timed exposure to 3 ¼M YK-4-279 as indicated (B) Colonies were counted using Nikon NIS Elements BR software. (* Denotes those plates which received 3 ¼M YK-4-279 treatment following replating.). This study was performed in triplicate and repeated two times.

We determined the replating efficiency of A4573 cells after 12–84 hours of treatment with 3 ¼M YK-4-279. Cells were treated for the indicated exposure time then replated without YK-4-279 to assay for colony formation (Figure 1A). Colony counts show that between the 24 and 36 hour time points there was a 3-fold decrease in colony growth (2229±101 to 84 ±100) and by 84 hours 25±12 colonies grew, representing a 98.9% drop from the 24 hour time point (Figure 1B). There were 53 and 25 colonies growing at 72 and 84 hours, respectively; however, when these colonies were harvested and replated in the continuous presence of 3 ¼M YK-4-279, no growth was observed (72* and 84*).

### Pharmacokinetic analysis demonstrates short plasma half-life of YK-4-279

To optimize dosing in animals, we determined the pharmacokinetics of YK-4-279 and its single enantiomers in Sprague-Dawly (SD) rats. Three rats of each gender were assigned to nine dose groups. Animals in Groups 1 through 3 received a single intravenous (IV) injection of racemic mixture, (S)-YK-4-279, or (R)-YK-4-279 at a dose of 25 mg/kg. Animals in Groups 4 through 9 received a single oral gavage administration of racemic mixture, (S)-YK-4-279, or (R)-YK-4-279 at a dosage level of 25 or 50 mg/kg. Racemic and (S)-YK-4-279 groups showed average elimination half-lives of 0.583 and 0.585 hours respectively; clearance averaged 2600 and 1260 mL/hour/kg for racemic and (S)-YK-4-279 respectively (Table 1). Clearance values were comparable between males and females, but were 2.0- to 2.2-fold higher after dosing with the racemic mixture compared to (S)-YK-4-279 (Table 1). However, given that approximately 50 percent of the racemic mixture is (S)-YK-4-279 and the clearance is inversely proportional to the dose, the IV clearance of (S)-YK-4-279 is considered to be similar whether a racemic mixture or only (S)-YK-4-279 is administered. Systemic exposure to (S)-YK-4-279 was evaluated using C_max_ and AUC values. C_max_ and AUC_8_ values were similar between males and females for a given dose group. With an expected enantiomer ratio of 1:1 in the racemic dose, Group 2 animals would have received a 2-fold higher dose of (S)-YK-4-279 than Group 1 animals. Consistent with this assumption, C_max_ and AUC_8_ values after a 25 mg/kg dose of only (S)-YK-4-279 were 2.1- and 2.0fold higher in males, respectively, and 2.3- and 2.2-fold higher in females, respectively, than those after a 25 mg/kg dose of the racemic mixture (Table 1). (R)-YK-4-279 was approximately 25% slower than the clearance of (S)-YK-4-279 (Supplemental table 1). Oral gavage with 25 or 50 mg/kg showed a modest initial increase in plasma concentrations following administration but plasma levels slowly decrease through the final 12-hour time point (Table 2). Absolute bioavailability of (S)-YK-4-279 following gavage administration, using cremophor/ethanol as an excipient, in SD rats was only 2–6% (Table 2).

**Table 1:** Pharmacokinetic values of (S)-YK-4-279 were obtained in SD rats following a single IV injection of racemic, (S)-YK-4-279 or (R)-YK-4-279

Treatment group (Target dose)	Gender	Observed	Cmax (¼M)	Terminal Elimination Half-Life (hr)	Clearance (mL/hr/kg)	AUClast (hr*¼M)	AUC∞ (hr*¼M)
Group 1: Racemic YK-4-279 (25 mg/kg)	Male (n=9)	39.9	0.564	2560	26.5	26.7
	Female (n=9)	37.1	0.602	2640	25.6	25.9
Group 2: (S)-YK-4-279 (25 mg/kg)	Male (n=9)	85.5	0.694	1300	52.4	52.4
	Female (n=9)	86.6	0.476	1220	56.0	56.0
Group 3: (R)-YK-4-279 (25 mg/kg)	Male (n=9)	BLOQ1	BLOQ	BLOQ	BLOQ	BLOQ
	Female (n=9)	BLOQ	BLOQ	BLOQ	BLOQ	BLOQ

1. BLOQ: below the limit of quantitation

**Table 2:** Pharmacokinetic values of (S)-YK-4-279 were obtained in SD rats following oral gavage administration of racemic, (S)-YK-4-279 or (R)-YK-4-179

Treatment group (Target dose)	Gender	Observed Cmax (¼M)	Observed Tmax (hr)	Terminal Elimination Half-Life (hr)	Apparent Clearance (mL/hr/kg)	AUClast (hr*¼M)	AUC∞ (hr*¼M)	Absolute Bioavailability (%)
Group 4: Racemic YK-4-279 (25 mg/kg)	Male (n=9)	0.142	1.00	3.84	90,300	0.563	0.754	2.83
	Female (n=9)	0.513	0.500	1.56	52,200	1.07	1.31	5.06
Group 5: (S)-YK-4-279 (25 mg/kg)	Male (n=9)	0.261	1.00	3.59	63,200	0.833	1.08	2.06
	Female (n=9)	0.620	2.00	1.86	30,000	2.14	2.28	4.07
Group 6: (R)-YK-4-279 (25 mg/kg)	Male (n=9)	BLOQ1	BLOQ	BLOQ	BLOQ	BLOQ	BLOQ	BLOQ
	Female (n=9)	BLOQ	BLOQ	BLOQ	BLOQ	BLOQ	BLOQ	BLOQ
Group 7 Racemic YK-4-279 (50 mg/kg)	Male (n=9)	0.241	2.00	4.23	116,000	1.04	1.18	2.21
	Female (n=9)	0.647	0.500	1.97	43,400	3.09	3.14	6.07
Group 8: (S)-YK-4-279 (50 mg/kg) Male (n=9)	0.380	2.00	3.94	55,400	1.78	2.46	2.35
	Female (n=9)	1.04	2.00	2.37	28,400	4.64	4.81	4.29
Group 9: (R)-YK-4-279 (50 mg/kg)	Male (n=9)	BLOQ	BLOQ	BLOQ	BLOQ	BLOQ	BLOQ	BLOQ
	Female (n=9)	BLOQ	BLOQ	BLOQ	BLOQ	BLOQ	BLOQ	BLOQ

1. BLOQ: below the limit of quantitation

**Figure 2:**
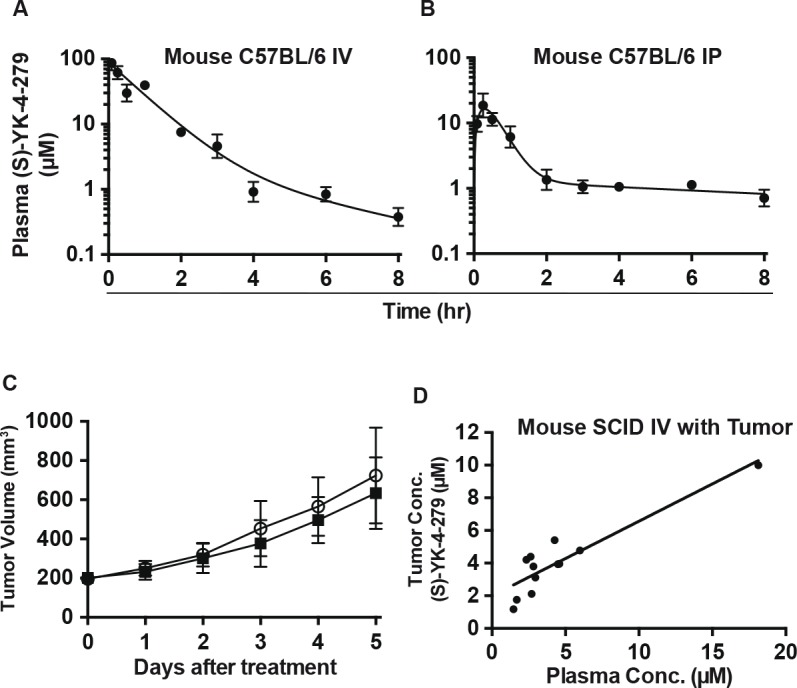
Pharmacokinetics of YK-4-279 in mouse models (A) Time course of (S)- YK-4-279 concentrations in male C57BL/6 mouse plasma following IV and (B) IP administration of 75 mg/kg racemic YK-4-279. (C) Six IV injections of 75 mg/kg/injection (S)-YK-4-279 for 5 Days did not show tumor regression in SCID mice (○ Control and ■ (S)-YK-4-279. (D) Plasma (S)-YK-4-279 levels correlate with xenograft tumor levels in SCID mice (R^2^=0.82543).

The poor oral bioavailability determined from the rat study caused us to focus on IV delivery. Since mice often metabolize compounds differently from rats [21], we also evaluated the intraperitoneal (IP) PK profle and compared it to the IV profile. We determined pharmacokinetics in C57BL/6 mice given IV (Figure 2A) or IP (Figure 2B) administrations of 75 mg/kg YK-4-279, which was the maximal solubility of compound in 20% Cremophor EL. While IP administration showed a steep rise in plasma concentration initially, (S)-YK-4-279 was substantially cleared leading to ~1 ¼M levels by 2 hours (Figure 2B). Absolute bioavailability of (S)-YK-4-279 following IP administration was 26%. In addition, drug levels in tumor were measured using SCID/bg mice with orthotopic xenografts that were treated with daily IV injections of (S)-YK-4-279 for 6 days. The pharmacokinetics demonstrated high peak levels that would exceed 3 ¼M for approximately 1 hour; thus, A4573 xenografts were treated with 75 mg/kg IV YK-4-279 QD. Consistent with the *in vitro* studies, tumors did not regress nor reduce their growth rate (Figure 2C). In order to determine (S)-YK-4-279 levels in the tumors, the mice were euthanized 2 hours following the fnal IV bolus dose for harvest of both tumors and plasma. The tumor tissue YK-4-279 concentration showed a positive correlation (r^2^ = 0.82) with plasma (ranged from 1.5–18 ¼M) levels (Figure 2D). Since the IV treatment was ineffective at reducing tumor growth, despite measurable levels in the tumor tissue, models of IP administration were used to design follow-up effcacy studies. In addition, various IP formulations were tested in order to enhance the bioavailability, yet none signifcantly improved absorption (Table 3).

**Table 3:** Plasma concentrations of (S)-YK-4-279 at 20 minutes following IP injection of 200 mg/kg of YK-4-279 (racemic mixture) in various formulations to C57BL/6 mice Five mice were used for each formulation, and mean (±SD) concentrations of (S)-YK-4-279 are shown in the table

Vehicle	% Lecithin	% DMSO	% Soybean Oil	(S)-YK-4-279 Concentrations (¼M)
10% w/v sucrose, 1% v/v Tween 80, 5% v/v ethanol, lecithin (variable), soybean oil (variable), DMSO (variable) in DI water	0.25	0	0	10.5 ± 8.6
	1.2	0	10	20.9 ± 22.8
	2.5	0	10	19.8 ± 20.5
	1.2	10	0	35.8 ± 45.0
	1.2	10	10	39.1 ± 54.6
	2.5	10	0	59.7 ± 58.6
	2.5	10	10	21.3 ± 11.3

### *In vivo* administration of YK-4-279 reduces xenograft growth

**Figure 3:**
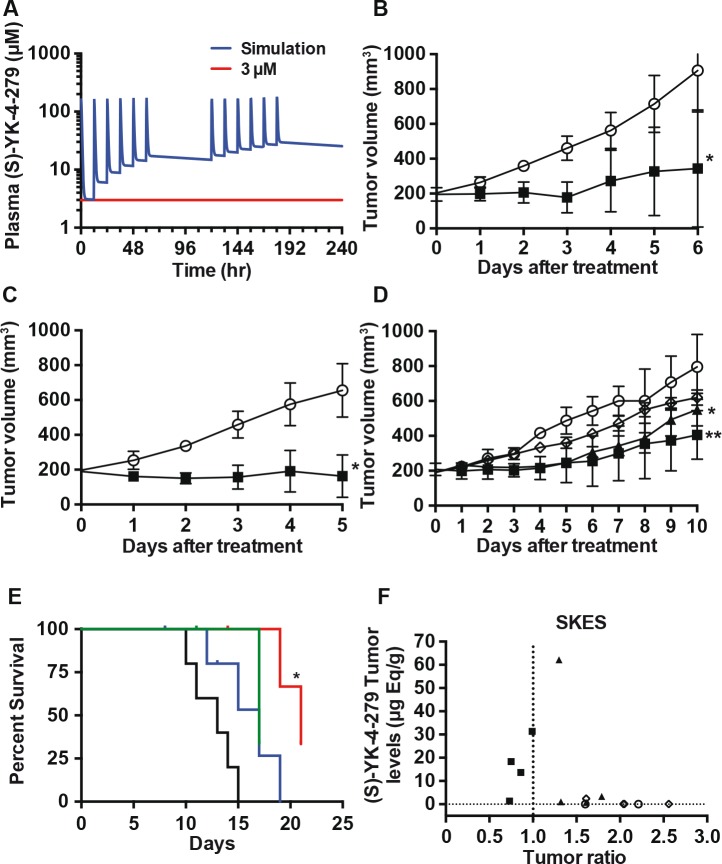
Inhibition of ES xenografts by YK-4-279 informed by pharmacokinetic dosing models (A) Plasma concentrations of (S)-YK-4-279 were simulated for a dosing regimen of BID IP injections of YK-4-279 (375 mg/kg) for 3 days followed by 2 days of no therapy. This cycle was repeated for another 5-day period, in order to maintain plasma concentrations of (S)-YK-4-279 greater than 3 ¼M. (B) A4573, (* p=0.04) or (C) SK-ES xenograft tumors were treated BID with IP injections of 400 mg/kg racemic YK-4-279 when tumors reached 200 mm^3^ (O Control and ■ racemic YK-4-279) (* p=0.02). (D) Mice with xenograft SK-ES tumors were treated BID with IP injections of 400 mg/kg racemic-, (S)-, or (R)-YK-4-279 over a 10-day period after tumors reached 200 mm^3^ (○ Control, ■ racemic YK-4-279, ▲ (S)-YK-4-279 and ◊ (R)-YK-4-279). ** Control vs. racemic, p=0.003, * control vs. (S)-yk-4-279 p=0.02. (E) Racemic YK-4-279 (red) treated SK-ES xenograft mice show survival benefit compared to those of control (black), (S)-YK-4-279 (green), and (R)-YK-4-279 (blue) treated mice. * Control vs. racemic p=0.013. (F) (S)-YK-4-279 xenograft tumor (SK-ES) tissue levels of racemic YK-4-279 treated mice showed higher (S)-YK-4-279 concentration than those of control, (S)-YK-4-279, (R)-YK-4-279 (◀ Control, ■ racemic YK-4-279, ▲(S)-YK-4-279 and ◊ (R)-YK-4-279).

A series of models that combined results from *in vitro* colony assays and *in vivo* PK data were developed to evaluate several potential dosing schemes for the mice (Supplementary Figures 1A-E). We selected an IP dosing strategy (twice daily (BID) injections of 375 mg/kg racemic mixture) that would maintain a 3 ¼M plasma levels for greater than 72 hours in the mice (Figure 3A). With this IP dosing regimen, xenograft growth of A4573 was 70% less in the YK-4-279 treatment group compared to vehicle control (Figure 3B, p=0.04). In addition, SK-ES xenograft mice also showed 70% less growth compared to the control group (Figure 3C, p=0.02). To confrm the impact of YK-4-279 treatment on xenograft growth, mice with SK-ES xenografts were treated with racemic, (S), or (R)-YK-4-279 until control tumors reached 1 cm^3^. Xenograft growth was reduced for those mice receiving both racemic and (S)-YK-4-279 over the control (Figure 3D, p=0.02 and p=0.003, respectively). Only the racemic YK-4-279 treatment led to improved overall survival (Figure 3E, p=0.013). Tumors were analyzed for drug levels of racemic, (R)-, and (S)-YK-4-279. Three of four mice treated with racemic compound had tumor levels of (S)-YK-4-279 that ranged from ~12-35 ¼g eq/g, which correlated with tumor growth ratios of less than 1.0, indicating response to the drug (Figure 3F). Those animals treated with the active (S)-YK-4-279 levels had tumor levels that were below 10 ¼g eq/g (Figure 3F). Tumor levels of (R)-YK-4-279 are also provided (Supplementary Figure 1F).

### YK-4-279 mouse xenograft confirms enantiospecific EWS-FLI1 targeted anti-tumor effect

**Figure 4:**
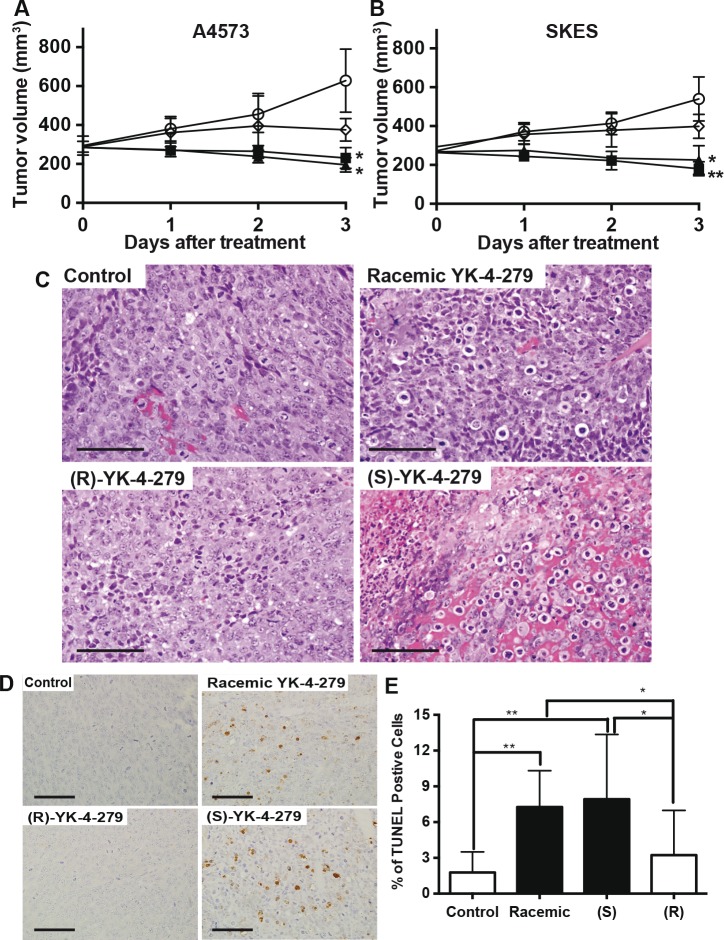
Racemic and (S)-YK-4-279 causes ES tqumor regression via apoptosis Mice with xenograft (A) A4573, * control vs. racemic p=0.008, * control vs. (S)-YK-4-279 p=0.005, and (B) SK-ES tumors were treated for 3 days BID with IP injections of vehicle control, racemic, (S)-, or (R)-YK-4-279 when tumors reached 200 mm^3^. ** Control vs. racemic p=0.003, * control vs. (S)-YK-4-279 p=0.006 (○ Control, ■ racemic YK-4-279, ▲ (S)-YK-4-279 and ◊ (R)-YK-4-279). (C) Examples of H&E staining from animals treated in (A). Scale bar = 50 ¼m. (D) TUNEL staining from animals treated in (A). Scale bar = 50 ¼m. (E) Quantifcation of TUNEL positive cells out of 500 counted on each slide from each tumor, ** control vs. racemic p=0.004, ** control vs. (S)-YK-4-279 p=0.002, * (R)-YK-4-279 vs. racemic p=0.03, * (R)-YK-4-279 vs. (S)-YK-4-279 p=0.01.

In order to evaluate tumors from animals treated in a similar fashion, including resection prior to central necrosis in controls, a 3-day, six-dose, experiment was executed. Treatment was initiated with racemic, (S)-, or (R)-YK-4-279 using BID IP administration at 400 mg/kg when tumors were well established at a volume of 250 to 300 mm^3^. Both ES models treated with either racemic or (S)-YK-4-279 regressed by 25–30% of tumor volume over 3 days, while tumors grew in control and (R)-YK-4-279 treated animals (Figures 4A and B). Two hours following the 6^th^ dose, mice were euthanized for tumor analysis. The DMSO control tumors, stained with hematoxylin and eosin, showed healthy mitotically active cells (Figure 4C), while those animals treated with racemic YK-4-279 (200 mg/kg (S) enantiomer) demonstrated vacuolar cell death and nuclear fragmentation consistent with apoptosis (Figure 4C). In the (S)-YK-4-279 treated animals (400 mg/kg), the cell death appeared to encompass a much greater amount of the A4573 tumor, consistent with a dosage effect over the racemic treatment (Figure 4C). The (R)-YK-4-279 treated tumors neither grew as fast as the DMSO control, nor regressed at the same rate as the (S)- or racemic tumors, which corresponded to their microscopic appearance of healthy tumor cells, but fewer mitotic figures than the DMSO control (Figure 4C). To confirm the apoptotic cell death, sections from A4573 tumors were stained with TUNEL (Figures 4D) and there was a 3 – 4 fold increase in TUNEL-positive cells treated with (S)-YK-4-279 compared to (R)-YK-R-279 treated tumors (Figure 4E). SK-ES tumors showed similar TUNEL results (Supplemental Figure 2A). Levels of each enantiomer were measured in tumor samples and (S)-YK-4-279 levels correlated with clinical regression (Supplemental Figure 2B–E).

**Figure 5:**
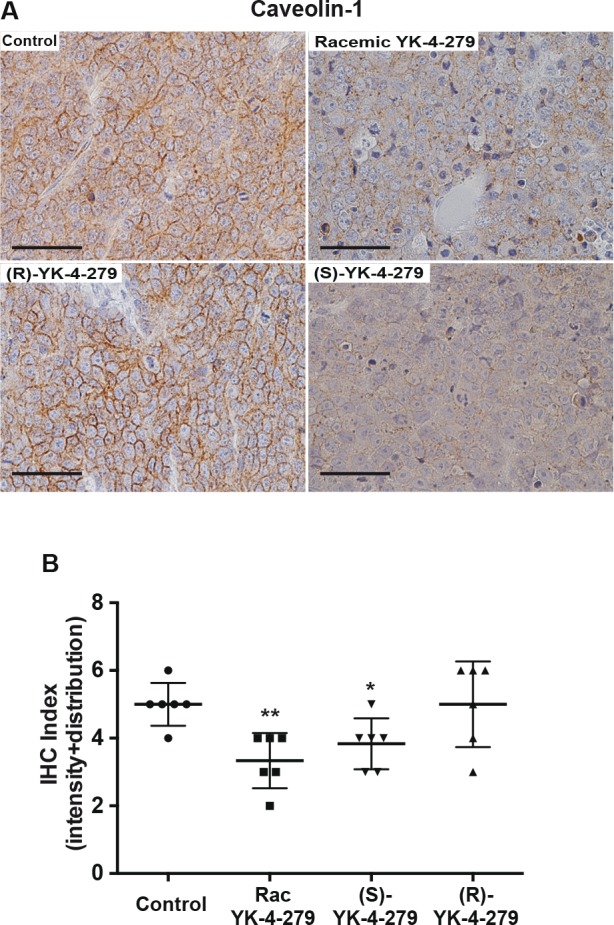
EWS-FLI1 target caveolin-1 expression reduced by racemic and (S)-YK-4-279 treated tumors (A) Representative examples of caveolin-1 IHC staining from animals treated in Figure 4. Scale bar = 50 ¼m. (B) Pathologist quantification of cavelolin-1 IHC in two blinded scoring sessions evaluating staining intensity and distribution, 0 – 3. The sum is reported in this graph, ** control vs. racemic p=0.004, * control vs. (S)-YK-4-279 p=0.04.

We evaluated the effect of EWS-FLI1 in animals treated with YK-4-279 upon caveolin-1, a recognized direct gene target whose expression is increased by EWS-FLI1 [22, 23]. Immunohistochemical staining of control and (R)-YK-4-279 treated animals shows expected membrane caveolin-1 expression (Figure 5A). Tumors from animals treated with racemic or (S)-YK-4-279 show reduced caveolin-1 staining. In addition, the staining pattern changes from membraneous to a more diffuse pattern (Figure 5A). Each slide was scored for intensity and distribution confirming significantly decreased caveolin-1 staining in racemic or (S)-YK-4-279 compared to the control animals (Figure 5B, p=0.004 and p=0.04, respectively).

### Continuous infusion (S)-YK-4-279 improves effcacy in novel rat xenograft model

The pharmacokinetic properties of (S)-YK-4-279 along with inability to deliver adequate dose over time through bolus injections in mice suggested a need for continuous infusion of compound. The solubility of (S)-YK-4-279 precluded the use of an osmotic pump, thus in order to deliver a continuous IV infusion, a larger animal xenograft was developed in the nude rat. A pilot study evaluated the tumor take of 4 ES standard xenograft cell lines (ES1, ES2, ES7, and EW8) while comparing orthotopic and fank subcutaneous injection. Animals received 5 million cells per site. Orthotopic injections of ES1 and ES7 demonstrated 5 of 5 animals with tumors, while ES2 and EW8 rates were 2 of 5 and 0 of 5 respectively (Supplemental Figure 3A). Subcutaneous injection led to very poor tumor growth showing 4 of 20 animals amongst all 4 cell lines leading to tumors (Supplemental Figure 3A).

**Figure 6:**
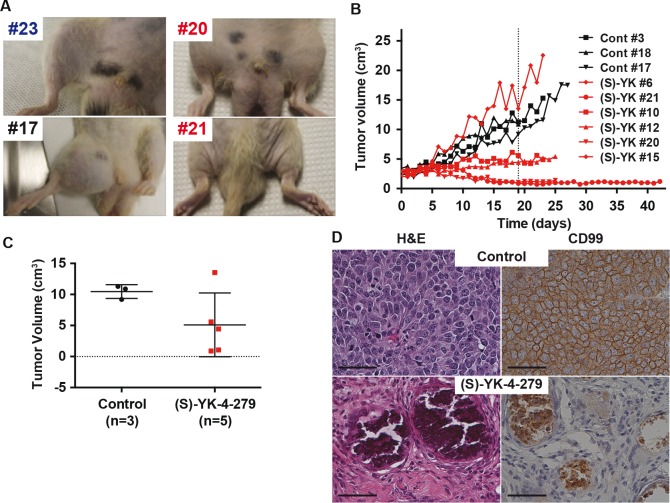
Rat orthotopic xenograft shows complete tumor regression with continuous infusion of (S)-YK-4-279 (A) Images of tumors at endpoint of study (blue: normal rats, black: control treated rats, red: (S)-YK-4-279 treated rats). (B) ES1 xenograft tumors were treated with continuous IV infusion of either control or 72 mg/kg/day (S)-YK-4-279 for 8 of 9 days per cycle (S)-YK-4-279 when tumors reached 2.5 cm^3^ in rats (Control: black and (S)-YK-4-279: red). (C) H&E and CD99 staining of rat xenograft with continuous IV treatment with control or 72 mg/kg (S)-YK-4-279 for 8 of 9 days per cycle. Scale bar = 50 ¼m. (D) Tumor volume on day 19 from start of treatment, control vs (S)-YK-4-279 p=0.08.

The efficacy study used only ES1 and ES7 cell lines with orthotopic injection. When animals had established tumors, as defined by two consecutive daily tumor volumes of greater than 2.5 cm^3^, they were randomly placed into vehicle control or continuous infusion treatment groups (72 mg/kg/day, 8 days on, 1 day off). The dose was established from pharmacokinetic modeling with a goal of maintaining 3 ¼M serum levels of (S)-YK-4-279. Animals received infusion via a tethered central venous catheter, which required mechanical adjustments to assure that animals could adequately eat and drink. Photographs show the hind legs of animals that did not grow a tumor (Figure 6A, #23), vehicle treated ES1 animals (Figure 6A, #17), or those ES1 tumors treated with (S)-YK-4-279 (Figure 6A, #20, 21). The growth curves for all ES1 animals show that two animals had complete regressions by tumor measurement, three animals had significantly less growth than controls, and one animal (#6) grew faster than the control animals (Figure 6B). A comparison of tumor size on Day 19 of treatment shows a significant difference between control and treated animals (Figure 6C, p=0.08). Representative pathology is shown (Figure 6D). In tumors that completely regressed, and did not regrow for over 24 days, the injection site did not show any evidence of ES cells (CD99 stain) at time of necropsy (Figure 6D). The ES7 tumor model also responded well to treatment, however, complete regressions were not seen (Supplemental Figure 3B).

## DISCUSSION

Inhibiting transcription factors derived from chromosomal translocations, that are well recognized as vital for the cancer, but here-to-fore designated ‘undruggable’ is a formidable challenge. These targets that lack enzymatic sites for classic site-directed inhibitors and are putatively intrinsically disordered proteins [10, 11, 24]. *In vitro* evaluation of a relatively small concentration of 3 ¼M of YK-4-279 was shown to be present for over 72 hours to get optimal cell killing. Thus, iterative pharmacokinetic simulations led to a need for continuous infusion dosing. These pharmacokinetic models were validated in a both ES xenograft mouse and rat studies that showed ES clinical tumor regression, molecular targeting, and apoptosis.

This work shows that tumor-specifc translocations can be successfully inhibited in an animal model with a small molecule. Intrinsically disordered proteins (IDP) are now recognized in many diseases as critical etiologic proteins and the biochemical properties of these proteins actually supports their being targeted by small molecules [25–27]. The small molecules that target the IDP are generally lower affinity for their target than enzyme inhibitors [18, 28–30]. In addition, proteinprotein interactions of IDP tend towards hydrophobicity. Thus small molecule protein-protein inhibitors will also be hydrophobic, therefore, lacking aqueous solubility. Finally, the mechanism of blocking protein interactions suggests that the small molecule inhibitors will need to be present for long enough time-scales to maintain protein separation for cell death to ensue. We chose to solve these challenges by using continuous infusion to block the interactions of IDP.

We measured differences in solubility between the enantiomers (data not shown), which may account for our comparative differences in animal studies between racemic YK-4-279 and (S)-YK-4-279. For example, a finding that requires further study is the correlation of plasma drug levels with that in the tumor tissue. The racemic-treated group demonstrated levels between 12 and 31 ¼g eq/g, and correlated with tumor inhibition. However, levels of (S)-YK-4-279 were significantly less than the expected two-fold increase based upon the dosage. This suggests formulation of the racemic may have improved solubility from the individual enantiomers, thus altering bioavailability [31, 32]. An alternative explanation is that one enantiomer affects the metabolism of the other, which is also a testable hypothesis, however our previously published data does not support this [20]. Alternative explanations for differences in (S)-YK-4-279 levels could be related to metabolism and require further evaluation.

*In vitro* evaluation of the enantiomeric biologic effects demonstrated that (S)-YK-4-279 was not only significantly more potent as an inhibitor, but also demonstrated significant specifcity upon the function of EWS-FLI1 compared to (R)-YK-4-279 [20]. There was a clear difference in the initiation of apoptosis with (S)-YK-4-279 compared to the (R)-YK-4-279. An observation however, with (R)-YK-4-279 treated animals showed a small growth retardation compared to controls, yet they did not die by apoptosis like the (S)-YK-4-279 treated animals. Pharmacokinetics did not show racemization of the compounds. We hypothesize that the (R)-YK-4-279 effect may be off target, as we have seen reduced growth in non-EWS-FLI1 expressing cell lines treated with YK-4-279, those cell lines also did not under go apoptosis when treated with (S)-YK-4-279 [20].

In order to support functional inhibition of EWS-FLI1 in an animal model, we were challenged by the heterogeneity that occurs when tumor cells die *in vivo*. Thus, assays in which tumors were ground-up and homogenized followed by qRT-PCR analysis were not successful in proving EWS-FLI1 target inhibition (data not shown). Using immunohistochemistry (IHC), we could evaluate individual tumor cells, including regional effects. However, a caveat of IHC is the requirement of specific antibodies, thus we selected caveolin-1 as a well-vetted target that would have a characteristic membrane staining pattern [22, 33]. Thus, in support of successful EWS-FLI1 targeting, caveolin-1 was reduced in animals treated with racemic or (S)-YK-4-279.

Efficacious clinical treatment of Ewing sarcoma relies heavily on compounds that will disrupt function of the primary protein driving oncogenesis, EWS-FLI1. Because YK-4-279 abrogates function of EWS-FLI1 by disrupting the interaction with a partner, RHA, adequate delivery of YK-4-279 to the tumor cells is imperative for its treatment course. In order to move this compound into the clinic, further investigation will be needed to optimize the formulation. In conclusion, this study supports continued preclinical development of YK-4-279 as a novel targeted therapy for patients with ES.

## MATERIALS AND METHODS

### Antibodies and Reagents

Antibodies obtained commercially: Caveolin-1 (Cell Signaling, #3238, Danvers, MA), CD99 (Abcam, #ab8855, Cambridge, MA). YK-4-279 synthesized by AMRI, Inc. (Albany, NY) and enantiomers (S)-YK-4-279, and (R)-YK-4-279 obtained from chiral separation of racemic compound.

### Time Point Re-plating Assay

A4573 cells were plated at a density of 2×10^5^ cells/dish in 60 mm dishes and incubated for 8 hours before treatment start with 3 ¼M racemic YK-4-279. Cells were removed with trypsin at their respective treatment time, cell suspensions were centrifuged, and cells were resuspended in 500 ¼l of media. One hundred microliters of cell suspension was plated in each 10 cm dish (triplicates) with 12 ml of media. Dishes were incubated for 1 week before staining with crystal violet stain. Colonies were counted using Nikon NIS Elements BR Software.

### Pharmacokinetic Analysis of YK-4-279 in Animal Models

Nine SD rats of each gender were assigned to nine dose groups. Groups 1 through 3 received a single IV injection of racemic mixture, (S)-YK-4-279, or (R)-YK-4-279 at a dose of 25 mg/kg. Groups 4 through 9 received a single gavage administration of racemic mixture, (S)-YK-4-279, or (R)-YK-4-279 at a dosage level of 25 or 50 mg/kg. Blood samples were collected from one animal per group per time point at target time points of pre-dose, 5, 15, 30 and 60 minutes, and 2, 4, 8 and 12 hours following dosing. Plasma was obtained from the samples and analyzed for (S) and (R)-YK-4-279 concentrations.

Ninety male C57/BL6 mice assigned to one of two dosage groups and were administered racemic YK-4-279 at a single dosage of 75 mg/kg by tail vein intravenous (IV) injection or IP administration. At a target of 5, 15, 30, and 60 minutes, and 2, 3, 4, 6, and 8 hours post-dose administration, blood specimens were collected from five mice per group per time point, processed to plasma, and stored at −70°C until analysis of (S)-YK-4-279 concentration.

### IV Efficacy Experiment

Twenty Fox Chase SCID beige mice per sex were inoculated with 2 million of A4573 cells. Tumor volume was monitored every day by caliper. After tumor size reaches 0.5 cm^3^, mice were randomized and received racemic YK-4-279 at a dosage of 25 mg/kg/administration by IV injection at 12-hour intervals through 60 hours following the first administration (T_0_, T_12_, T_24_, T_36_, T_48_, and T_60_). At a target of 2 hours following the last YK-4-279 administration, the mice were humanely euthanized. Blood was collected from four mice per sex and processed to plasma, while tumor tissue was snap-frozen and stored at −70°C until analysis of YK-4-279 concentration.

### Orthotopic Mouse Xenograft Model

Two million A4573 or SK-ES Ewing's sarcoma cells in 0.1 mL were injected into an orthotopic paraosseous location, adjacent to the left proximal tibia, in 5-week-old female severe combined immunodeficient-beige (SCID/bg) mice (Harlan Laboratories, Inc., Indianapolis, IN). After primary tumors reached 250 – 300 mm^3^ size, mice were randomized and received intraperitoneal injection (IP) with vehicle control (0.25% Lecithin solution or DMSO), (S)-YK-4-279, (R)-YK-4-279 or racemic YK-4-279 at a dose of 400 mg/kg twice a day (BID) for 7 days in a week. The tumor volume was determined by the formula (D × d2/6) × p, where D was the longer diameter and d was the shorter diameter. Tumor volume was monitored every day by caliper until the tumor size reaches 1cm^3^. Mice were euthanized and primary tumors were collected. For the pharmacodynamics study mice were randomized and received 6 doses treatment with vehicle control (0.25% Lecithin solution or DMSO), (S)-YK-4-279, (R)-YK-4-279 or racemic YK-4-279 at a dose of 400 mg/kg twice a day (BID). Mice were euthanized after 2 hours of last 6^th^ dosing and primary tumors were collected. Animal studies were approved by an Institutional Animal Care and Use Committee of the Georgetown University.

### Continuous Infusion Rat Xenograft Model

Six million ES1 or ES7 Ewing sarcoma cells in 0.2 mL were injected into an orthotopic paraosseous location, adjacent to the left proximal tibia, in 6-week-old male nude rats (Charles River, Burlington, MA). After primary tumors reached 2.0 cm^3^ size, vascular catheters were surgically placed and rats were allowed to recover. Rats were randomized and received continuous IV infusion of either vehicle control (15% cremophor EL) or (S)-YK-4-279 was begun following two consecutive tumor measurements of greater than 2.5 cm^3^. Dose administration of vehicle control or (S)-YK4-279 for each of the three dosing cycles consist of a single bolus injection of 4 mg/kg and 8 days of 9 days continuous infusion at a dose 2.5 mg/ml and a target rate 5 ¼l/min (72 mg/kg/day) to produce a steady state plasma drug level of 3 ¼M. The tumor volume was determined by the formula (D × d2/6) × p, where D was the longer diameter and d was the shorter diameter. Tumor volume was monitored every day by caliper until the tumor size reaches 20 cm^3^. Rats were euthanized and primary tumors were collected. Animal studies were approved by an Institutional Animal Care and Use Committee of the Battelle.

### Determination of YK-4-279 Concentrations and PK Analysis

The plasma and tumor sample analysis was performed using calibration standards and Quality Control (QC) samples prepared in control rat or mouse plasma and tumor tissues. Plasma and tumor calibration standards were prepared from stock solutions of YK-4-279. The calibration standards, blanks, QC samples, and study samples were then processed by solid-liquid extraction (SLE) followed by analysis using liquid chromatography with mass spectrometry (LC-MS). The LC-MS systems used for the analysis of YK-4-279 was composed of Shimadzo prominence pump and auto-sampler (Kyoto, Japan), a Sciex API 5000 MS (Toronto, Ontario), with turbo ion spary in the positive ion mode. The samples for the analysis of total YK-4-279 were injected into a 50 × 2 mm Gemini- NX, 5 ¼m column (Phenomenex, Torrance, CA). The samples for the analysis of the specific enantiomers of YK-4-279 were injected into a 150 × 2 mm Lux Cellulose-2, 3 ¼m Phenomenex column. The transitions monitored were 366 amu to 135 amu for YK-4-279 and 369 amu to 138 amu for the YK-4-279-d_3_ internal standard. YK-4-279 concentrations were calculated using peak area response ratios and a regression equation constructed from the concentrations and peak area response ratios of the calibration standards. The lower limit of quantitation was 5.0 ng/mL in plasma and 5.0 ¼g/g in tumor tissue. The concentration-time profiles were evaluated by compartmental modeling and non-compartmental analysis using WinNonlin (Pharsight Corporation, Mountain View, CA). The PK parameters obtained from the compartmental modeling were used in simulations to identify optimal dose level and dosing schedule for maintaining plasma concentration of (S)-YK-4-279 above 3 ¼M.

### Histology, IHC and Slide Evaluation

All tumor tissues were fxed for a minimum of 24 hours in 10% neutral buffered formalin, dehydrated through a graded series of alcohols, cleared in xylenes, infiltrated with paraffin wax and embedded in wax molds. Tissue sections were cut at 5 microns and placed onto Superfrost Plus charged slides (Fisher Scientific, Pittsburgh, PA). Hematoxylin and Eosin (Leica Microsystem Inc., Buffalo Grove, IL) staining was performed on a Leica Autostainer XL.

Five micron sections from formalin fxed paraffin embedded tissues were de-paraffinized with xylenes and rehydrated through a graded alcohol series. Heat induced epitope retrieval was performed by immersing the tissue sections at 98°C for 20 minutes in 10 mM citrate buffer (pH 6.0) with 0.05% Tween. IHC staining was performed using the VectaStain Kit from Vector Labs (Burlingame, CA) according to manufacturer's instructions. The sections were evaluated, in blinded fashion two times, by a pathologist (B.K.).

### Statistical Analysis

Xenograft tumor growth study used the unpaired t test with Welch's correction (Figure 3B and C). For xenograft tumor growth study and calveolin-1 IHC index used ordinary one-way ANOVA (Figure 3D, Figure 4A and B, Figure 5B). Survival was estimated with the Kaplan-Meier method and unstratified log-rank statistical analysis to test for differences, with pairwise comparison between groups (Figure 3E). All statistical tests were two-tailed. Tissue IHC experiments were conducted in duplicate. In vitro studies were validated in triplicate experiments.

## Supplementary Figures


