# Supplementary material for: Pharmacokinetic modeling optimizes inhibition of the ‘undruggable’ EWS-FLI1 transcription factor in Ewing Sarcoma
Source: Oncotarget. 2013 Nov 11;5(2):338–50. doi: 10.18632/oncotarget.1495 (PMC3964211; doi:10.18632/oncotarget.1495)
Supplement: Supplementary file 1 [file oncotarget-05-338-s001.pdf]

Pharmacokinetic modeling optimizes inhibition of the 'undruggable' EWS-FLI1 transcription factor in Ewing Sarcoma - Hong et al

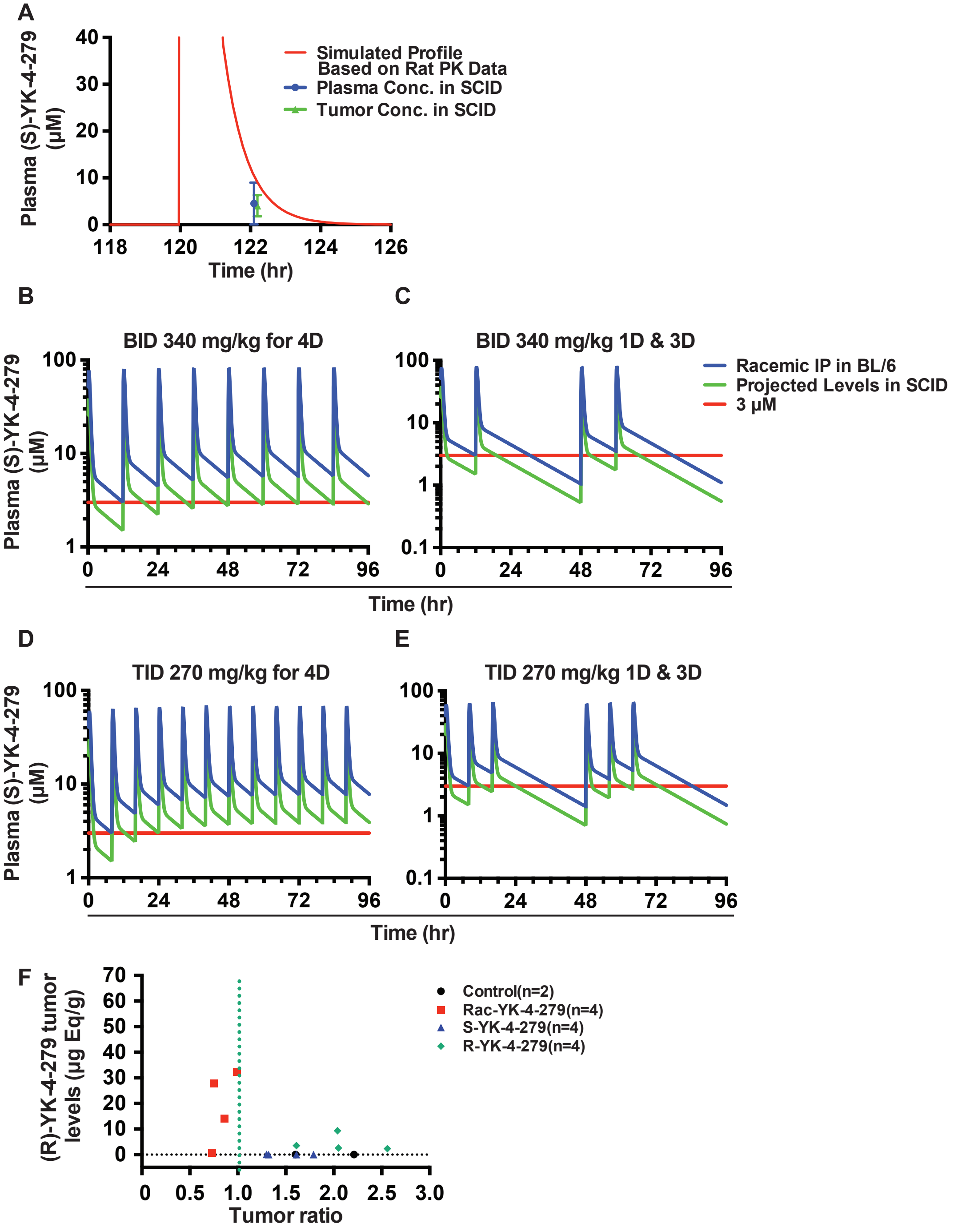

Supplemental Figure 1: YK-4-279 dose and timing simulations to achieve target plasma levels (3 µM) in rat and mouse models. (A) Simulated profile of (S)-YK-4-279 levels based on rat PK data is correlated with plasma levels and xenograft tumor levels in SCID mice after 5 days treatment. (B) Simulated PK profiles for (S)-YK-4-279 concentrations in plasma following different IP dosing regimens in C57BL/6 and SCID mice (BID 340 mg/kg IP for 4 days), (C) BID 340 mg/kg IP on Day 1 and 3, (D) TID 270 mg/kg IP for 4 days, (E) TID 270 mg/kg IP on Day 1 and 3. WinNonlin software by Pharsight Corporation of Sunnyvale, CA was used for pharmacokinetic simulations. (F) (R)-YK-4-279 xenograft tumor (SK-ES) tissues levels of racemic YK-4-279 treated mice showed higher (R)-YK-4-279 concentration than those of control, (S)-YK-4-279, (R)-YK-4-279 (○ Control (black), ■ racemic YK-4-279 (red), ▲ (S)-YK-4-279 (blue) and ◇ (R)-YK-4-279 (green)).

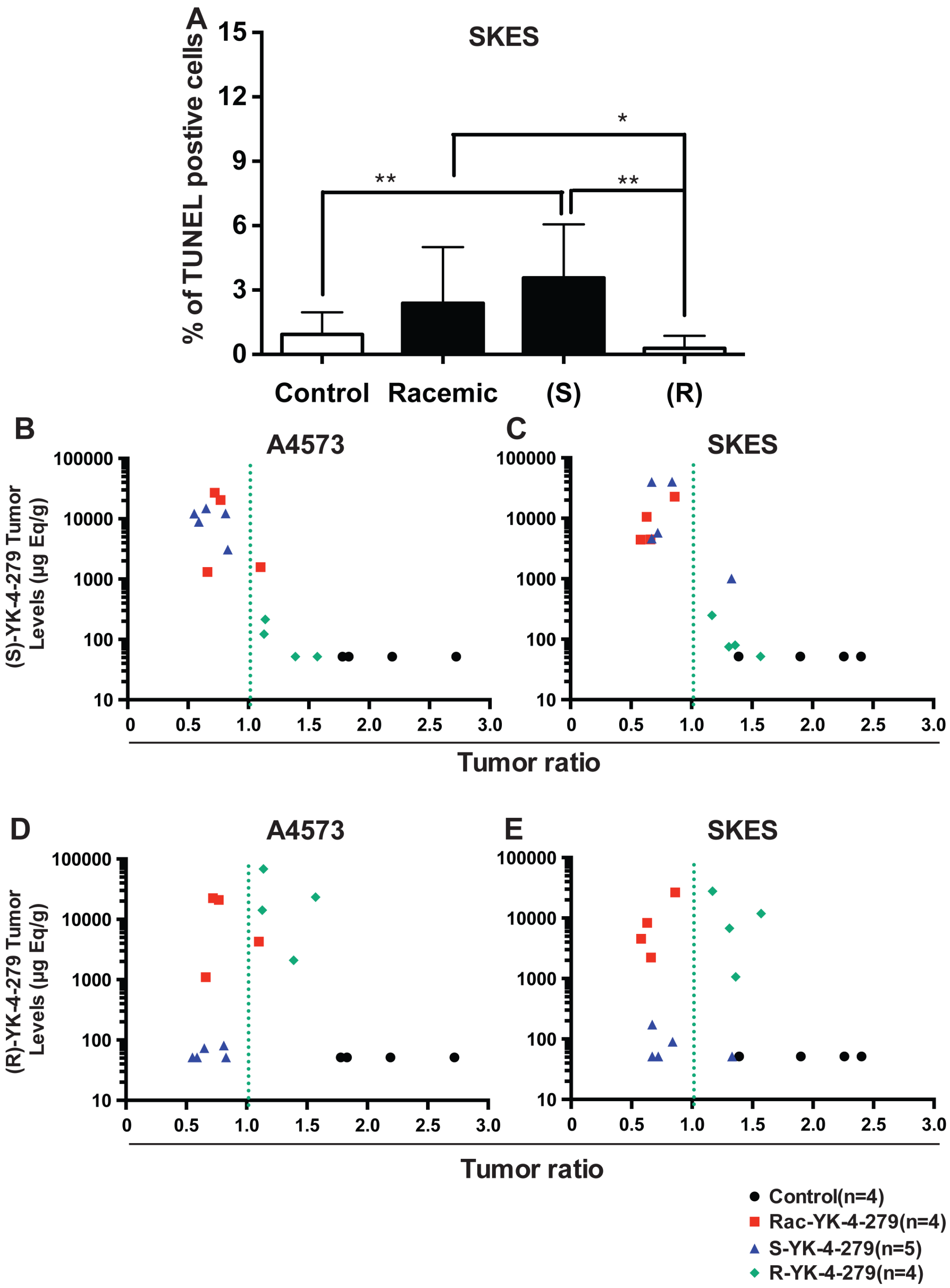

Supplemental Figure 2: Racemic and (S)-YK-4-279 treated xenograft tumor tissues show high (S)-YK-4-279 concentration and racemic and (S)-YK-4-279 induce apoptosis in tumor cells in SK-ES xenograft mice. (A) Mice with SK-ES xenograft tumors were treated for 3 days BID with IP injections of vehicle control, racemic YK-4-279, ** control vs. (S)-YK-4-279 p=0.005, * (R)-YK-4-279 vs. racemic p=0.04, ** (R)-YK-4-279 vs. (S)-YK-4-279 p=0.0006. In (B) A4573 or (C) SK-ES (S)-YK-4-279 xenograft tumor tissue levels of racemic YK-4-279 and (S)-YK-4-279 treated mice showed higher (S)-YK-4-279 concentration than those of control and (R)-YK-4-279 (○ Control (black), ■ racemic YK-4-279 (red), ▲ (S)-YK-4-279 (blue) and ◇ (R)-YK-4-279 (green)). In (D) A4573 or (E) SK-ES (R)-YK-4-279 xenograft tumor tissue levels of racemic YK-4-279 and (R)-YK-4-279 treated mice showed higher (R)-YK-4-279 concentration than those of control and (S)-YK-4-279 (○ Control (black), ■ racemic YK-4-279 (red), ▲ (S)-YK-4-279 (blue) and ◇ (R)-YK-4-279 (green)).

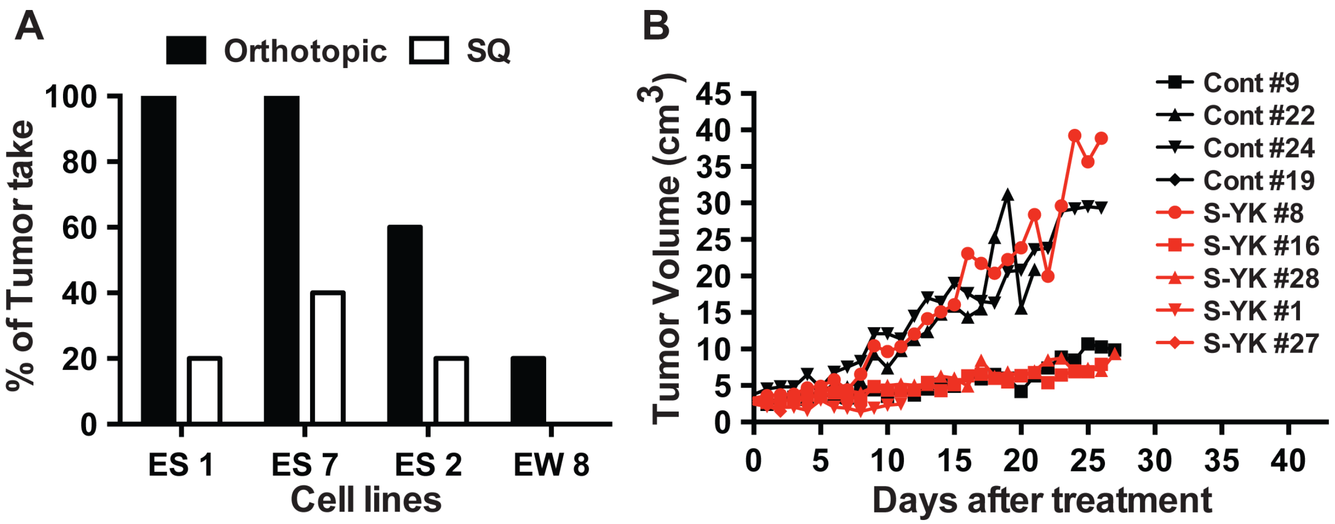

Supplemental Figure 3: Rat orthotopic xenograft shows complete tumor regression with continuous infusion of (S)-YK-4-279 and higher (S)-YK-4-279 concentration in ES xenograft tumor tissues. (A) Orthotopic xenograft of 4 different human Ewing Sarcoma cells shows 100% tumor take rate compare to SQ injection in rat. (B) ES7 xenograft tumors were treated with continuous IV infusion of either control or 72 mg/kg/day (S)-YK-4-279 for 8 of 9 days per cycle (S)-YK-4-279 when tumors reached 2.5 cm^3 in rats (control: black and (S)-YK-4-279: red).
